# Metabolic syndrome, dyslipidemia, hypertension and type 2 diabetes in youth: from diagnosis to treatment

**DOI:** 10.1186/1758-5996-2-55

**Published:** 2010-08-18

**Authors:** Alfredo Halpern, Marcio C Mancini, Maria Eliane C Magalhães, Mauro Fisberg, Rosana Radominski, Marcelo C Bertolami, Adriana Bertolami, Maria Edna de Melo, Maria Teresa Zanella, Marcia S Queiroz, Marcia Nery

**Affiliations:** 1 Group of Obesity and Metabolic Syndrome, Endocrinology and Metabolism Service, Hospital das Clínicas da Faculdade de Medicina, São Paulo University (HC-FMUSP). Av. Dr. Enéas de Carvalho Aguiar, 155 - 8º andar - bloco 3. São Paulo, Brazil; 2 Arterial Hypertension and Lipids Sector of Hospital Universitário Pedro Ernesto - State University of Rio de Janeiro (UERJ). Rua São Francisco Xavier, 524. Rio de Janeiro, Brazil; 3 Adolescent Center, Department of Pediatrics, Federal University of Sao Paulo (UNIFESP). Rua Pedro de Toledo, 650, 2o andar. São Paulo, Brazil; 4 Endocrinology and Metabolism Service of Hospital de Clínicas, Department of Nutrition, Federal University of Paraná (UFPR). Rua General Carneiro, 181. Curitiba, Brazil; 5 Dante Pazzanese Institute of Cardiology of the São Paulo State Health Department. Av. Dr. Dante Pazzanese, 500. São Paulo, Brazil; 6 Service of Endocrinology, Department of Medicine, Federal University of São Paulo (UNIFESP). Rua Pedro de Toledo, 650, 2º andar. São Paulo, Brazil; 7 Group of Diabetes, Endocrinology and Metabolism Service, Hospital das Clínicas da Faculdade de Medicina, São Paulo University (HC-FMUSP). Av. Dr. Enéas de Carvalho Aguiar, 155 - 8º andar - bloco 3. São Paulo, Brazil

## Abstract

Overweight and obesity in youth is a worldwide public health problem. Overweight and obesity in childhood and adolescents have a substantial effect upon many systems, resulting in clinical conditions such as metabolic syndrome, early atherosclerosis, dyslipidemia, hypertension and type 2 diabetes (T2D). Obesity and the type of body fat distribution are still the core aspects of insulin resistance and seem to be the physiopathologic links common to metabolic syndrome, cardiovascular disease and T2D. The earlier the appearance of the clustering of risk factors and the higher the time of exposure, the greater will be the chance of developing coronary disease with a more severe endpoint. The age when the event may occur seems to be related to the presence and aggregation of risk factors throughout life.

The treatment in this age-group is non pharmacological and aims at promoting changes in lifestyle. However, pharmacological treatments are indicated in special situations.

The major goals in dietary treatments are not only limited to weight loss, but also to an improvement in the quality of life. Modification of risk factors associated to comorbidities, personal satisfaction of the child or adolescent and trying to establish healthy life habits from an early age are also important. There is a continuous debate on the best possible exercise to do, for children or adolescents, in order to lose weight. The prescription of physical activity to children and adolescents requires extensive integrated work among multidisciplinary teams, patients and their families, in order to reach therapeutic success.

The most important conclusion drawn from this symposium was that if the growing prevalence of overweight and obesity continues at this pace, the result will be a population of children and adolescents with metabolic syndrome. This would lead to high mortality rates in young adults, changing the current increasing trend of worldwide longevity. Government actions and a better understanding of the causes of this problem must be implemented worldwide, by aiming at the prevention of obesity in children and adolescents.

## Introduction

The worldwide concept of metabolic syndrome in children and adolescents is still a matter of discussion mainly because studies on this age group are scarce. In this Symposium of Metabolic Syndrome, Dyslipidemia, Hypertension and type 2 Diabetes in Children and Adolescents, organized by the Department of Metabolic Syndrome of the Brazilian Society of Diabetes, many aspects of those clinical conditions were widely discussed, covering diagnosis, early atherosclerotic lesions, therapeutic management and non-pharmacological as well pharmacological treatment. The increased risk of development of comorbidities like dyslipidemia, arterial hypertension and glucose intolerance were also discussed and the following question was pointed out for the therapeutic management of each condition: When should we treat these youngsters pharmacologically?

More than 150 health care professionals including physicians, nurses and dietitians attended this Symposium which took place in São Paulo, providing a forum for debate on this important issue.

## Metabolic Syndrome Diagnosis in Children and Adolescents

Several large epidemiology cohort studies have documented that obesity and metabolic syndrome are associated with cardiovascular outcomes in adults, such as myocardial infarction, cerebrovascular disease and sudden death [1]. In recent years, there has been a greater concern about the presence of obesity and metabolic syndrome in children and adolescents [2]. Upper Obesity is the core aspect of insulin resistance and seems to be the physiopathologic link common to metabolic syndrome.

There is no consensus regarding the diagnosis of metabolic syndrome in children and adolescents as recently discussed by Mancini [3]. It is evident that each component of the syndrome must be identified as early as possible in order to prevent definitive lesions. The question is how to do this and which cut-offs must be adopted for this diagnosis.

The diagnosis of metabolic syndrome in children and adolescents requires the assessment of the abdominal circumference (or BMI), blood pressure, lipoproteins and glycemia. While the value of waist circumference as a measure of visceral adiposity remains somewhat debated, an accepted measurement of waist circumference by percentiles was established [4]. Nevertheless, there is some controversy about how to measure the abdominal circumference in children and adolescents. There are some proposals: one of which was published in 1999 by Freedman - one of the authors of the Bogalusa study [4]. In this study the authors correlated the 90^th ^percentile of abdominal circumference with increased levels of LDL cholesterol, glycemia, insulin and diminished HDL levels. Limits were established and the use of a table for abdominal circumference was proposed (above the 90^th ^percentile, considered to be the maximum normal limit).

Some people claim that metabolic syndrome in children must be defined by BMI and not by circumference. An adjusted BMI curve according to gender and age was proposed to the Brazilian population, produced by the group supervised by Monteiro & Conde from the School of Public Health of the University of Sao Paulo [5]. Despite the existence of this curve, even for the matter of comparing studies, BMI curves adjusted by the North-American CDC have been more widely used [6]. Normal BMI varies according to the child's age. Thus, it is impossible to apply a BMI range of 18.5-24.9 to a 12 or 13-year-old child. Overweight is defined as above the 85^th ^percentile and obesity above the 95^th ^percentile (tables available at http://www.cdc.gov). Nonetheless, with the launching of the WHO references for children 5-19 years old, obesity is considered as above the 97^th ^percentile or 2^nd ^SD or z score (available at http://www.who.int/growthref).

### Proposals for the Definition of Metabolic Syndrome in Children and Adolescents

For a discussion about the various proposals for the definition of metabolic syndrome in children, refer to reference 3.

The definition considered most appropriate and which was added to this SBD publication is the one proposed by the IDF. It divided children into age groups. There was not a well defined proposal for children under 6 years of age, due to the lack of data. Unlike the criteria presented above, for a matter of convenience, the cut-offs in this proposal were fixed for pressure, lipids and glucose, and abdominal circumference points were assessed by percentile. In children aged 6-10, the cut-offs for metabolic and blood pressure variables were not well defined, therefore, only adiposity levels were evaluated (considering abdominal circumference above the 90^th ^percentile). The same criteria would be used for children aged 10-16; regarding glucose and lipid metabolism, fasting glucose levels ≥100 mg/dl, triglycerides ≥150 mg/dl, HDL cholesterol below 40 mg/dl or the use lipid lowering drugs were considered risk factors as well blood pressure ≥130 or ≥85 mmHg. If the patient has abnormal abdominal circumference and two more factors, the metabolic syndrome diagnosis is established. The difference is that, for adolescents over 16 years of age, there is a distincition between HDL ≤ 40 for men or ≤ 50 for women, as described in Table 1.

**Table 1:** Classification of metabolic syndrome in children and adolescents [3]

Criteria/components	Age		
	
	6 to < 10 years-old	10 to 16 years-old	> 16 years-old
Adiposity definition	WC ≥ 90^th ^percentile	WC ≥ 90^th ^percentile	WC ≥ 90 cm (boys) or ≥ 80 cm (girls)
Glucose metabolism	Without cut-off definition for MS diagnosis	Fasting blood glucose ≥ 100 mg/dl	Fasting blood glucose ≥ 100 mg/dl
Dyslipidemia	Without cut-off definition for MS diagnosis	Tg ≥ 150 mg/dl or HDL-ch ≥ 40 mg/dl or taking LLD	Tg ≥ 150 mg/dl or HDL-ch ≥ 40 (boys) or ≥ 50 mg/dl (girls) or taking LLD
Arterial hypertension	Without cut-off definition for MS diagnosis	DBP ≥ 130 or SBP ≥ 85 mmHg or taking AHD	DBP ≥ 130 or SBP ≥ 85 mmHg or taking AHD

WC: waist circumference; MS: metabolic syndrome; Tg: triglyceride levels; HDL-ch: HDL-cholesterol levels; LLD: lipid-lowering drug; DBP: diastolic blood pressure; SBP: systolic blood pressure; AHD: antihypertensive drug.

The IDF criteria, though more convenient, fail to include some children in the diagnosis of Metabolic Syndrome. On the other hand, it would be easier to accept as it does not use multiple tables to assess several anthropometric and metabolic criteria [7].

## Atherosclerosis in Young Adults

The acknowledgement of the presence of atherosclerotic disease in young adults was demonstrated in necropsy studies of American soldiers who were victims of the Vietnam War. The lesions found in these individuals varied from the presence of foam cells and fatty stretch marks to type 4 lesions, i.e., mature atherosclerotic plaques, with a well developed lipid core and the presence of cholesterol and inflammatory cell crystals [8]. Based on these findings and on the findings of other studies, it was possible to certify that young individuals may already present advanced atherosclerotic lesions. Studies then continued to even lower age ranges, such as children and adolescents, where atherosclerotic evidence was also demonstrated. In this scenario, the *Bogalusa Study *stands out: approximately 12,000 children and adolescents were prospectively and longitudinally followed up. The authors analyzed the prevalence of the main cardiovascular risk factors (RF) and observed that they recurred throughout life, in a phenomenon known as "tracking", i.e., if they were present during early age, they were more likely to persist throughout life [9]. About 190 of these individuals died from different causes and were submitted to necropsy, and the presence of fibrous plaques in the aorta and coronary arteries was described, confirming that atherosclerosis was present since a very early age. Researchers also found that lesions were increasingly severe according to more advanced age and in the presence and/or aggregation of cardiovascular risk factors. Among present risk factors in this cohort, obesity and systolic (but not diastolic) blood pressure stood out, either alone or combined, as the most correlated factors to the presence of aortic and coronary atherosclerotic lesions. The majority of complex lesions were associated with total cholesterol levels, LDL levels and less intensely with triglyceride levels. Smoking was also an important determining factor of early and accelerated development of atherosclerotic disease in these individuals [10]. Another study, also conducted with children and adolescents, the *Pathobiological Determinants of Atherosclerosis in Youth *(PDAY) also demonstrated that the aggregation of cardiovascular risk factors increased the risk of developing atherosclerotic disease, detected on the intimal vessel surface during necropsy studies [11].

These findings motivated researchers to conduct longitudinal studies on children and adolescents in an attempt to detect the prevalence of the different cardiovascular risk factors. Among these, the *Estudo do Rio de Janeiro *has been following up a cohort of over 3000 students of both genders, aged 6-15, for over 17 years, aiming at building blood pressure (BP) normality curves for such individuals. The prevalence of the different cardiovascular risk factors was analyzed in a subgroup made up of 365 children and adolescents and it was found to be 14.6% for arterial hypertension, 21.6% for overweight and/or obesity, 39.6% for dyslipidemia and 14.8% for smoking [12]. Similar studies conducted in the USA reported prevalence percentiles of 5-20% for hypertension, 20-33% for overweight/obesity, 13-35% for dyslipidemia and 25-35% for smoking [13]. Remarkably, such prevalence is very similar to that found in adults.

The continuum concept presented by Dzau et al. advocates that cardiovascular disease seems to develop slowly from early stages until the clinical outcome, which is frequently lethal. The earlier the appearance of cardiovascular risk factors and the greater the time of exposure, the higher the chance of developing the disease and the more severe the final endpoint is. The age at which the event may occur seems to be related to the presence and aggregation of cardiovascular risk factors in the course of life [14].

The aggregation of several cardiovascular risk factors in the same individual has been exhaustively demonstrated in all age ranges. Miura et al. showed the association between blood pressure and mortality from atherosclerotic disease in young adults in a 30-year follow up study. The authors observed that, for each 10 mmHg increase in systolic blood pressure, there was a 1.5% increase in mortality [15]. More recently, McCarron et al. demonstrated that cardiovascular risk doubled for each 20-mmHg increase in systolic blood pressure and for each 10-mmHg in diastolic blood pressure [16].

Once the problem is identified, the crucial concern is about which procedure needs to be adopted with a child who has cardiovascular risk factors There are still few intervention studies about this age range and the general recommendation is to intensify efforts in order to correct risk factors and/or the presence of comorbidities with non-pharmacological measures. There is no long term study which confirms the safety of prolonged drug use, in this age range, for the treatment of hypertension, dyslipidemia, obesity and diabetes among others. However, considering the individual risk of children, pharmacological treatment has been frequently recommended in special situations which will be discussed later in this symposium.

## Nutritional Guidance for Children and Adolescents with Metabolic Syndrome

The increasing prevalence of overweight and obesity in youngsters has also been observed in Brazil. A recent National study, the *Nutri Brasil Infância*, conducted with 12 universities in the country showed that over 23% of Brazilian children present excess weight up to the age of five - a fact that previously would have only be seen in later age groups [17,18]. We have observed an important increase in sedentary lifestyle and low physical activity levels in adolescents attending both public and private schools. It is important to emphasize that Brazilian children in general do not reach 30% of the amount of moderate physical activity time per week suggested by the World Health Organization, which would be 150 minutes [19].

Considering non-pharmacological interventions for treating children and adolescents with MS, promoting lifestyle changes, which include increasing physical activity, eating balanced diets and engaging in psychosocial approaches must be the initial step [20]. It is easier to control meals at schools than at home. The main problem regarding diet control is how to reach a healthy weight during a phase of such fast growth and gain of both fat and lean mass. The major goals in dietary treatments are not only limited to weight loss, but also to an improvement in the quality of life, with the modification of risk factors associated to comorbidities, personal satisfaction of the child or adolescent and trying to establish healthy life habits from an early age. This is not a simple task, especially when our children live among families with completely inadequate lifestyles and eating habits [21]. Some goals that are well established for adults will depend on age range, puberty stage and gender when applied to children. For example, a 5 to 10% weight reduction, which is an exceptionally important criterion of treatment success for adults, may not be adequate for a child in growth and development, especially in puberty. Regarding diets, the general goal is to promote weight loss or stabilize the rate of weight gain, reduce visceral fat, correct dyslipidemia and normalize blood pressure [22]. These diets are generally rich in fiber and low in saturated fat, cholesterol and plain sugar content. Several types of diet allow weight loss and the reduction of blood pressure levels, and consequently minimize cardiovascular events [22]. However, the major challenge is how to adjust these diets to such a young population with habits which are so difficult to change, who often follow the family's standard of inadequateness.

From a practical point of view, it is recommended that food with high energy density be avoided, which promotes the reduction of macronutrient imbalance. Some discrepant aspects have been shown recently in our field, such as avoiding milk and dairy products, recommended by some groups. This is a conduct that contradicts epidemiological evidence which relates greater calcium intake to weight loss and a lower incidence of cardiovascular events [23,24].

In 2007 [25], the American Medical Association drafted a number of new measures and attempted to resolve MS from the dietary and behavioral standpoint, by establishing four phases of treatment:

### Phase 1

global orientation for the child and the family. Simple measures such as encouraging meals like breakfast and others with the family could be useful. It is suggested that meals outside of the home be limited and family meals be encouraged, in order to allow the child to reach self-regulation, moderating the use of restrictive behaviors. In this phase, the goal would be to reduce the rate of weight gain, thereby favoring growth. If the child did not evolve properly, phase 2 would be the next suitable step.

### Phase 2

structured weight management programs designed by trained primary health care professionals. These groups would work with balanced diet plans, limiting high energy density foods in the diets. In this phase, the goal is to improve the structure of meals and snacks, improve structured physical activity to at least 60 minutes per day, and reduce daily TV, video-game or computer time to one hour or less. Data from research and opinion institutes show that the use of electronic devices by children in Brazil has reached an average of 4.8 hours per day. The family needs to be monitored in all behavioral aspects. The objective of this period is obviously to reduce the BMI (body mass index). However, a weight loss standard is still being determined: 450 g per month for 2 to 11-year-olds, climbing up to almost 1 kg for children and adolescents above 11 years old. If no favorable result is seen at this point, the next step would be phase 3.

### Phase 3

Implementation of activities which include the monitoring of food and physical activity, as well as short-term goals for the diet, physical activity and change in behavior must be performed with the help of a multidisciplinary team of specialists in childhood obesity, professionals with experience in giving orientation about diet and physical activity, offering the proper psychological, educational and emotional support. For children who remain above the 95^th ^percentile, with comorbidities and who have not been successfully treated in the previous phases, this phase must be tried. In this phase, diet counseling is required beyond the general orientation: a more intense restriction on calorie intake is established in association with an increase in continuous physical activity. Additional strategies are established, such as the use of food replacements, very low calorie diets, and later the use of medications - leaving gastric bypass surgery as a last possible intervention. Goals for children of ages from 6 to 11, especially in overweight children, are to maintain the weight until there is a possibility of recovery, or to get BMI rate to decrease. In obese children, we should have a lower average of weight loss. In severely obese individuals, weight loss still cannot be higher than 1 kg per week, which is a common barrier in most phase 3 healthcare programs. However, we have to evaluate if these children who enter programs of fat use restriction, carbohydrate intake modification and most importantly, highly restrictive weigh loss counseling may present excessive weight loss in the future, which could later result in eating disorders and premature bulimia or anorexia.

In older children, an even greater caloric restriction can be seen especially for the morbidly obese, considered as those above the 99^th ^percentile, or in other words the third standard deviation or third Z score. It is important to emphasize, that for children and adolescents to reach a lifestyle change with nutritional education and physical activity; it must be an ongoing effort that should be started at an early age. We should think that, without the adoption of dietary measures and the implementation of change in the whole family's life style, the chance of reducing factors associated with metabolic syndrome in children and adolescents will be very small.

## Physical Activity in Children and Adolescents - A Realistic View

The increase in sedentary life over the last few decades is closely linked to the increase in the prevalence of overweight and obesity both in adults and children [26]. A survey conducted in London [27,28] has shown that people walked 63 miles less in 2003 compared to 1975, indicating a significant reduction in energy consumption, which contributes considerably to weight gain in adults as well as in children.

Regarding TV viewing, it has already been demonstrated that watching television, regardless of age, increases BMI, mainly in the group that watched TV for over three hours a day [29]. A key concern is that, in general, watching television is associated with higher food consumption. A study conducted in Brazil has shown that, during the times of day when children watch television, advertisings are fundamentally about the group of fats, sugars and sweets; therefore, children are encouraged to ingest high calorie foods [30]. An epidemiological assessment conducted with Hispanic Americans in Houston, Texas, has shown that obese and thin children and adolescents had low physical conditioning, which was even lower in the obese and in girls [31]. Another interesting fact shown by this study was that physical activities are different among boys (more intense activities) and girls (light and moderate activities). Physical activity changes from birth up to 18 years of age, and in general, girls practice less physical activity than boys, which is probably related to worse results in terms of weight loss with physical activity in women [32].

Regarding cardiovascular risk factors related to excess weight and metabolic syndrome in children and adolescents, different studies have shown that, at any age, the number of risk factors is inversely related to physical activity and to physical conditioning [33,34].

A meta-analysis was conducted with a systematic review of over 600 studies: out of the 14 selected with supervised exercises, four compared aerobic activity to controls, nine compared the effects of diet therapy and behavior therapy with and without aerobic and resistance exercises. Weight reduction of about 2.7 kg was observed, favorable to combined intervention, and up to 4 kg, when it was higher in intensity and length [35].

The regular practice of exercise results in a change in body composition and several physiological and metabolic benefits. In a study conducted in São Paulo, a one-year intervention with physical activity and diet in obese adolescents, visceral fat loss was observed over time both in boys and in girls [36]. In Curitiba, a study group assessed the influence of dietary counseling and physical activity (three times a week), over a 24-week period on 64 obese adolescents. The study demonstrated a reduction in BMI and in waist circumference in both girls and boys, but with greater responses in boys. In obese adolescents with metabolic syndrome, there was also an improvement in cardiovascular risk factors such as a decrease in blood pressure, triglyceride levels, glucose levels and in insulin resistance (determined by HOMA-R), as well as an increase in HDL-cholesterol levels [37]. It was also noted in this study that, the reduction in insulin resistance was associated to weight loss. However, even in adolescents who did not lose weight, there was an improvement in insulin sensitivity through physical activity, probably resulting from metabolic conditioning. Physical activity is able to improve insulin sensitivity in a single exercise session, due to the increase in glucose uptake by the muscle, but it returns to the initial levels 48 hours after finishing the exercise. In order to obtain proper metabolic conditioning, exercise must be practiced regularly [38].

In addition to reducing risk factors, exercise promotes changes in other factors. It improves endothelial function; decreases proinflammatory cytokine levels; reduces stress levels in adults, children and adolescents; preserves lean muscle mass in weight loss programs and improves appetite control in previously sedentary individuals[39,40].

There is ongoing debate about the best exercise for children or adolescents to lose weight. In this age range, specific recommendations for physical activity - type, intensity, length and frequency - vary widely. The prescribed exercise type and "dosage" for a certain individual will depend on the goal to be reached, i.e., losing weight, controlling comorbidities, physical conditioning, preventing obesity and weight recovery [41].

It has been suggested that in order to obtain beneficial health effects, mainly in order to reduce cardiovascular risk factors, school-age children should participate in daily moderate to intense physical activities, for 60 minutes or more, as well as reducing sedentary behavior to less than two hours a day [42,43]. However, several studies have shown that both obese children and adolescents respond well to 30-40 minutes of physical activity, 3 times a week, improving cardiovascular conditioning and the metabolic syndrome components such as hypertriglyceridemia, hyperinsulinemia and arterial hypertension, even if weight loss is minimal, as long as the intervention is continuous and combined with good dietary counseling [44,45]. The most indicated exercise for weight loss and to reduce metabolic changes is aerobic exercise (jogging, swimming, cycling and other leisure activities [42].

Physicians must pay attention, as adolescents change their mind all the time, and they must practice the type of physical activity they decide to, and switch it whenever they want. Very often, patients do not want or do not like doing exercise. It is necessary to identify the restraints which keep adolescents from participating in activities [46-48]. It is important to emphasize to patients and their family that exercise does not have to be competitive, and that the main goal is to improve physical conditioning, to reduce weight and comorbidities. The compliance to physical exercise, at this age range is the same as in adults, i.e., 20 to 80%. Frequently, obese children or adolescents have low physical conditioning. Their perception of effort is higher than the perception of thin individuals, and skeletal muscle pains are common, as a result of excess weight [49]. Therefore, it is important to establish progressive goals (mainly regarding intensity and volume of activities) until each patient meets the goals assigned to them. Encouraging them to keep a diary recording the number of hours spent watching TV, at the computer or playing games and the frequency of physical activity also helps the follow-up process. Obesity is a chronic disease which affects individuals of all age ranges and is difficult to handle. Lifestyle changes are essential for the treatment to succeed. Among such changes, reducing sedentary life and practicing regular physical activity stand out, mainly during childhood and adolescence, when therapeutic pharmacological options are difficult.

The prescription of physical activity to children and adolescents requires extensive integrated work among multidisciplinary teams, patients and their families, in order to reach therapeutic success.

## Pharmacological Treatment of Obesity in Children and Adolescents

The pharmacological treatment of obesity in children and adolescents has as its main difficulty in the fact that studies showing evidence about most drugs available in the market are lacking for such age group. At present, the drugs available for the treatment of obesity are: sibutramine, orlistat and catecholaminergic agents (diethylpropione, fenproporex and mazindol) [50] The choice criterion for which drugs to treat obesity with at such an age range follows the same standards suggested for the treatment of adults. In the case of food compulsion or lack of satiety, sibutramine is the most indicated drug [51]. Orlistat is more frequently indicated when there is excessive food intake, including high-fat diets. In order to use this drug, the absence of severe gastrointestinal disease must be confirmed [52]. Regarding catecholaminergic agents, there is no global experience, not even in adults, with relevant scientific studies. It can be indicated for attention-deficit disorder [50], which is not uncommon in obese children, because diethylpropione and fenproporex have structural similarities to methylphenidate, the drug of choice for this disorder. Thus, when it comes to obese children with attention-deficit disorder, rather good results may be obtained with diethylpropione, fenproporex and even mazindol, although it is a drug with no phenylethylamine nucleus. Topiramate is a drug which has already been tested for obesity [53], with good results, but due to side effects has not been released in the market for such a purpose. However, it has been increasingly used, mainly on individuals with mood disorders and food compulsion and it is important to emphasize that it is contraindicated for patients with a history of kidney stones. Bupropion is one of the drugs of choice for tackling tobacco addiction, but it may also work as an anti-obesity drug [54], although contraindicated for epileptics. Fluoxetine is a serotonin reuptake inhibitor which may help treat obesity [3], particularly when associated with arterial hypertension or sleep apnea. Relative contraindications are bipolar disorders and epilepsy. It can be inferred that the drug choice will depend on the patient's personality, behavior, dietary habits and associated diseases [55].

There are a few studies with metformin (a drug which increases hepatic insulin sensitivity) in obese non-diabetic and insulin-resistant adolescents, demonstrating that it may be a useful drug to fight obesity in children [56-58]. The longest was a year-long study which demonstrated reduced BMI and insulin resistance measured by HOMA-R in 28 obese adolescents with a dose of 2 g of metformin per day [58].

In an orlistat placebo-controlled study which included 539 obese adolescents, followed for 12 weeks, it was observed that associated risk factors improved and there was variable weight loss, up to 15 kg in some cases, with reduced insulin levels [7]. A 12-week study [59] also showed important results in all associated comorbidities; on the other hand, the concern with gastrointestinal effects in children seems unjustified, as according to such studies and to our experience, they do not seem to be more severe than in adults. The weight-independent effects of orlistat are described as improvements in cholesterol and hepatic enzyme levels, lower increments of postprandial triglyceride levels and increased insulin sensitivity [9].

Two important sibutramine studies have been conducted with obese adolescents and published in literature. One of them was conducted in Brazil [60] for 12 weeks and demonstrated important results in weight reduction when compared to placebo. The other study included 498 obese adolescents, followed for 12 months, and showed equally important weight-related results and co-morbidity improvements with sibutramine [61]. Its side effects in adolescents were the same and at a similar proportion to events reported by adults [51].

Taking into account the studies so far including children and adolescents, the use of metformin, orlistat and sibutramine is indicated starting from 10 years of age (for type 2 diabetes treatment), 12 years of age and 16 years of age (for obesity treatment), respectively.

However, this does not mean that pharmacological treatment must not be used in lower age groups. In severe cases of obesity, when health risks are extremely evident, the aim is to prevent early co-morbidity and mortality.

## Dyslipidemia

Dyslipidemia in children and adolescents has become a frequent clinical condition, especially due to the increase in overweight and obesity prevalence in this age range [62]. Treatment depends on the type of dyslipidemia, i.e., if the cause is familial, with clinical signals such as xanthomas or if dyslipidemia is inserted in the global picture of metabolic syndrome. The lipid profile is completely diverse: while in familial dyslipidemia there is significant hypercholesterolemia with LDL-c levels possibly over 400 mg/dl [63] in dyslipidemia associated with metabolic syndrome, there is a higher trend of hypertriglyceridemia, low HDL-c levels, and LDL-c levels without quantitative but rather qualitative changes, such as small dense particles which are more atherogenic [64]. Dyslipidemia at this age range may have a genetic component with or without the environmental component, such as improper diet, familial hypercholesterolemia, familial combined hyperlipidemia and hypertriglyceridemia, and there may be types of dyslipidemia which are predominantly environment-related, but which may also have a genetic component, and in such cases excess weight or weight gain is extremely important [64].

### Prognosis

The prognosis depends on the type of dyslipidemia, and it is well established that hypercholesterolemia, in its homozygotic form, is extremely severe [65] with a prevalence of 1/1 million. Male children usually of 15 years of age may already present a coronary event or death. The heterozygotic form has a prevalence of 1/500 [63]. Women have a better evolution with longer life expectancy than men, most of whom have already been diagnosed with coronary disease by the age of 50 years old [63].

For dyslipidemia associated with metabolic syndrome, recent data [63] have shown that the presence of metabolic syndrome during childhood is associated with the development of coronary disease 25 years later, i.e., in adulthood [66].

### Lipid Profile Assessment

When should the lipid profile of children and adolescents be assessed? The 2005 1^st ^Guideline for Atherosclerosis Prevention in Children and Adolescents [67] recommends that every child over 10 years old assesses their cholesterol levels (Table 2). It is still being discussed if the most indicated assessment would be by capillary blood (simple trial) or by total venous blood (to determine the full lipid profile). Such recommendations are more important when children present any of the risk factors listed below:

**Table 2:** Recommended lipid levels in adolescents up to 19 years old

Lipides	Desired (mg/dl)	Coterminous (mg/dl)	Increased (mg/dl)
TC (mg/dl)	< 150	150 - 169	≥ 170
LDL-c (mg/dl)	< 100	100 - 129	≥ 130
HDL-c (mg/dl)	≥ 45		
TG (mg/dl)	< 100	100-129	≥ 130

TC: total cholesterol; LDL-c: LDL-cholesterol; HDL-c: HDL-cholesterol; TG: triglycerides.

1. Family history of early atherosclerosis in parents, siblings or grandparents (before 55 years old in men and before 65 years old in women);

2. If parents have cholesterol levels > 240 mg/dl;

3. If children have other associated risk factors, such as hypertension, obesity and saturated fat or trans fatty acid rich diet;

4. Children who have other diseases which may be associated with dyslipidemia, such as AIDS and hypothyroidism;

5. Children who use drugs which may predispose them to dyslipidemia, e.g., isotretinoin for acne;

6. Children who present clinical manifestations of dyslipidemia, even if they are not pathognomonic, such as xanthoma and/or xanthelasma - they are strong indicators - and occasionally the presence of repeated pancreatitis in children who have hypertriglyceridemia.

The new recently published American Guideline in Pediatrics [68] brings some important differences regarding treatment:

1. Always initiate the treatment with non-pharmacological measures;

2. Age of starting pharmacological treatment reduced from 10 to 8 years old;

3. If the child is healthy and does not present other problems, the LDL-c level limit for pharmacological treatment is 190 mg/dl;

4. If the child has important family history or two risk factors, the LDL-c level limit decreases to 160 mg/dl;

5. If the child is diabetic, the limit LDL-c level is 130 mg/dl

However, the early pharmacological treatment motivated an editorial published in New England [69]. The author demonstrates concern with the use of drugs in situations that could be reverted by intensifying lifestyle changes.

### Classification of Familial Hypercholesterolemia

Although most of this review focuses on metabolic syndrome, we also need address hypercholesterolemia in children.

Several mutations in several locations could result in familial hypercholesterolemia, such as: mutation in the LDL receptor [10], mutation in Apo B-100 [70] and mutation in protease PCSK-9, which destroys LDL receptors decreasing their half-life [70]. A certain mutation of such protease would result in longer receptor half-life with lower prevalence of coronary disease [71]. Recently, such a mutation was identified in black and Caucasian populations, but the effect observed in the black population was more intense. They have LDL-c levels approximately 30% lower than those observed in the average New York population; at the same time, they presented 88% lower cardiovascular mortality rates. Considering the pharmacological intervention studies, for each mg/dl of LDL-c level reduction observed with the treatment, it is possible to decrease the prevalence of coronary disease by about 1%. Thus, we may infer that in this NY population, the lowest mortality rate was superior to the expectations of the lowest LDL-c levels [71]. It is believed [72] that such a difference is due to the fact that the low LDL-c levels observed in this population have been the same in these individuals since birth, different from the treatment with statins which was usually initiated in clinical studies, in adulthood.

### Diagnosis of Familial Hypercholesterolemia

For the diagnosis of familial hypercholesterolemia, two non-exclusive but complementary strategies may be used: 1) Cascade trial - which in selected cases are researched due to personal and/or family history. In a case of hypercholesterolemia or early coronary disease (men under 55 years old and women under 65 years old), descendants and family members are called for a lipid profile assessment. In such cases, the new American Guideline already recommends the trial in one-year-olds [68]. 2) Generalized trial - all children are assessed. Considering the difficulties in obtaining the genetic diagnosis, if children present LDL-c levels over 95 percent for their age range and gender and if there is family history of early coronary disease, the diagnosis is probably familial hypercholesterolemia[68], and the treatment is often initiated immediately, especially in cases of tendinous xanthoma.

### Treatment

1. Non-pharmacological, including diet, physical activity and phytosterols.

2. Pharmacological

Cholestyramine has always been accepted as the first-line therapy drug for children due to it being a resin which is not absorbed in the intestines; its effect is intestinal, not systemic. It may affect the absorption of liposoluble vitamins, which may result in important side effects in children. The main side effect presented is intestinal obstipation, therefore it is better tolerated by children than by the elderly [73]. Cholestyramine has a reduced effect in decreasing LDL-c levels (< 19%), as every intestinal-acting drug tends to have a diminished effect due to intestinal adjusting (when cholesterol absorption decreases in the intestine, a chylomicron particle with lower cholesterol levels is generated and as it reaches the liver, it stimulates a higher expression of the LDL receptor in order to increase cholesterol reception) [73].

The 2008 Pediatrics Guideline already determines statin as the first-line drug for treating hypercholesterolemic children other than cholestyiramine [68]. The drug ezetimibe acts specifically in cholesterol absorption, decreasing LDL-c levels by 20%, further decreasing it when associated with statin[74]; it may also be used alone or in association.

Statins have been used in adults since 1987, but long-term studies still need to be conducted regarding its prescription to children and adolescents in order to determine the safety of using it over 20 or 30 years and if its continued use is associated with the improvement of clinical endpoints [13]. Side effects are similar to those observed in adults, and there has been no evidence of change in the growth, sexual and cognitive development curves [75-77], as the decrease in cholesterol synthesis does not compromise hormonal synthesis or brain development [78].

Using fibrate and nicotinic acid in children and adolescents is recommended only in exceptional situations, as with fenofibrate, which is used when non-pharmacological measures are ineffective at treating hypertriglyceridemia [73]. However, further studies must still be conducted regarding the use of these drugs for such an age range [68].

Using statin presents other benefits, such as the improvement in flow-dependant endothelial function [79] and the improvement in the progression of the carotid intima-media thickness in individuals with familial hypercholesterolemia. A study conducted in the Netherlands demonstrated that the treatment of familial hypercholesterolemia, when initiated at 10 years old, changes the perspective of individuals [78].

It is concluded that, for dyslipidemia in children and adolescents, family history and all the risk factors must be assessed in order to determine the best therapeutic strategy which allow the goals established for their age range to be reached, thus preventing clinical cardiovascular endpoints.

## Hypertension in Children and Adolescents

The global increase in the prevalence of obesity, which has also affected children and adolescents, is favoring an increase in blood pressure and therefore, the prevalence of arterial hypertension [80]. The impact of weight gain on blood pressure is not the same in all age ranges nor is it between men and women. In the young male population, divided into quartiles according to body mass index (BMI), an increase in the prevalence of arterial hypertension can already be seen in the second quartile. The association of BMI and hypertension is greater among men below 45 years of age and in this subpopulation, 60% of the hypertension cases can be attributed to excess weight.

In women under 45 years old, the impact of weight gain on the prevalence of arterial hypertension is lower, compared to men. Above this age however, an evident association between BMI and hypertension prevalence has been observed. It is likely that in women above 45 years old or in menopause, weight gain associated with the accumulation of abdominal fat favors the increased prevalence of hypertension.

### Physiopathological Mechanisms Associated to Arterial Hypertension in Metabolic Syndrome

The possible mechanisms that trigger arterial hypertension associated with metabolic syndrome are mainly related to the presence of visceral fat, which results in insulin resistance. The subsequent hyperinsulinemia promotes the increase in sodium absorption by the kidneys, which favors arterial hypertension [81]. In addition, hyperinsulinemia also increases the sympathetic activity, which contributes to the development of arterial hypertension [81].The accumulation of visceral fat elevates the activity in the renin-angiotensin system, due to an increased production of angiotensinogen, which consequently favors arterial hypertension [82,83]. Experimental studies have yet indicated that leptin could be another factor within the physiopathology of arterial hypertension as it causes the sympathetic activity to increase [84].

### Arterial Hypertension Diagnosis in Children and Adolescents

Arterial hypertension should be measured in every child as of three years old, mainly when there are family risk factors. The method of choice is auscultation. The child should be in a sitting position and at ease, preferably leaning against the backrest and with their feet supported; the right arm should be at the same level as the heart and blood pressure should be taken on at least three different occasions. The diagnosis of hypertension if blood pressure is altered in these three occasions was established. Systolic pressure is determined by the first Korotkoff sound and the diastolic by the fifth sound. It is important to notice the size and the location of the cuff, which should cover 40% of the circumference of the arm, mid length between the acromion and the olecranon, and the stethoscope should be placed in the cubital fossa. These recommendations are important for measuring blood pressure adequately. The normal levels of blood pressure are based on gender, age and percentile of height and are presented in tables which can be used to determine whether the individual is hypertensive or normotensive [85]. Some programs are already available on the Internet. By inserting the child's age and height, as well as systolic and diastolic blood pressure, the information on the percentile the child is in regarding arterial blood pressure is obtained, which enables the determination of whether the child is hypertensive or not.

Arterial Blood Pressure Monitoring in children and adolescents shows an increase in systolic blood pressure with age. Daytime blood pressure is more elevated than nighttime blood pressure. Other similar studies have shown that systolic blood pressure varies much more than diastolic blood pressure with reference to age.

Considering that blood pressure changes constantly throughout childhood and that there are modifications of normal values depending on gender, age and height, normal blood pressure in children is defined as systolic or diastolic blood pressure below the 90^th ^percentile [85].

Pre-hypertension is defined when the child presents blood pressure between the 90^th ^and 95^th ^percentiles, or in adolescents when blood pressure is greater than 120/80 mmHg. Stage 1 of arterial hypertension is defined when the child presents blood pressure between the 95^th ^and 99^th ^percentile with the addition of 5 mmHg, and stage 2 when the pressure levels are above the 99^th ^percentile with the addition of 5 mmHg [85].

An increase in blood pressure is associated with lesions of target organs, which has been shown in the Rio de Janeiro Study. This study demonstrated an association of BMI, increase in blood pressure and hypertrophy in the left ventricle, measured either in grams or by the left ventricular mass index [86]. The comparison between the hypertensive group (blood pressure scores above the 95^th ^percentile) and normotensive group (blood pressure values below the 50^th ^percentile) showed a significant difference in the left ventricle mass in these adolescents. A third group which presented variable blood pressure, sometimes above the 95^th ^percentile and sometimes below these values, presented intermediary left ventricle mass which indicates that alterations in target organs, from very early stages of blood pressure level elevation, can already be observed.

### Treatment

It is important to remember that arterial hypertension in children and adolescents, even when associated with obesity, demands investigation for the diagnosis of secondary hypertension because the younger the child and the more elevated the levels of blood pressure, the greater the chance of developing secondary hypertension.

In the pre-hypertension phase (between the 90^th ^and 95^th ^percentiles, or blood pressure above 120/80 mmHg, but lower than the 95^th ^percentile in adolescents), the recommendation is lifestyle [87] changes which promote weight loss and the improvement of insulin sensitivity.

Drug treatment is indicated for stage 1 hypertension, or upon the presence of symptoms (headaches or lightheadedness) or when there is no response to the immediate lifestyle changes for a period of at least six months, especially when there is a family history of hypertension or premature cardiovascular disease [85]. The goal of the therapy is to obtain blood pressure levels below the 95^th ^percentile. However, in the presence of target organ lesions or other cardiovascular risk factors - like type 1 diabetes (T1D) with nephropathy or even T2D - the goal is to obtain levels below the 90^th ^percentile.

In stage 2 hypertension, the goals are similar to those described for stage 1. The treatment should also aim at reducing cardiovascular morbi-mortality in the long run, although no studies conducted with children show that arterial hypertension treatment will result in morbi-mortality reduction.

There is no consensus regarding the best pharmacological agent to be used when initiating essential hypertension therapy in children. It is suggested that the smaller dose be used, enough to be effective and that it does not cause side effects. In choosing anti-hypertensive drugs however, we should preferably choose those which will not enhance insulin resistance, promote weight gain (beta-blockers), or worsen glucose intolerance (thiazide diuretics) or the lipid profile, and that are able to control blood pressure for 24 hours. The presence of other cardiovascular risk factors should also be considered such as microalbuminuria, which reflects a systemic endothelial lesion not restricted to the glomerulus, as well as a hypertrophy of the left ventricle.

Lifestyle change measures should always be implemented as early as possible, as it is easier for children to change life habits. A controlled consumption of salt from the early months of life is recommended, fighting excess weight from the first year of life and always encouraging the premature practice of physical activity, along with healthy eating habits.

In drug treatments of hypertension associated with metabolic syndrome, the mechanisms which generate hypertension such as the increase in activity of the renin-angiotensin system and sympathetic system as well as the increase of sodium absorption, should be interfered with if possible always with the aim of decreasing cardiovascular morbi-mortality. The drugs more widely used and their mechanisms of action are listed below:

1. Angiotensin receptor blockers and converting enzyme inhibitors (ACEIs) inhibit the renin-angiotensin system and reduce cardiovascular mortality in adults. In the ACEI class, the drugs suited for pediatric use are benazepril, captopril, enalapril [88], fosinopril[89], lisinopril [90] and quinapril. These drugs are totally contraindicated during pregnancy and, thus, when used by women or teenage girls of fertile age, they must be accompanied by the use of safe contraceptive methods. It is always necessary to evaluate the levels of potassium and creatinine during therapy and coughing is a frequent side effect. The FDA in the United States has approved the use of ACEIs in children over six years old and for individuals with creatinine depuration above 30 ml/min, as this condition offers a lesser risk of hyperkalemia. These drugs present advantages, when used on adults, for individuals with insulin resistance, as they are associated to a lower risk of diabetes onset [91]. The angiotensin II receptor blockers (ARBs) approved for use by children are losartan and ibesartan. These are also contraindicated during pregnancy and recommendations are similar to doses described for ACEIs. Losartan has the advantage of being available in suspension formulation, although not in Brazil. The use of ARBs can also be associated with a lower incidence of diabetes, and these agents can be used for long periods, as they are very well tolerated, due to their effects being similar to placebo.

2. Calcium channel blockers (CCBs) promote natriuresis and have a vasodilating action; amlodipine, felodipine, isradipine and slow-release nifedipine are approved for pediatric use. Amlodipine and isradipine are available, in the United States, in suspension formulation, which favors its use by children.

3. Beta-blockers have been recommended for young patients for a long time and the agents bisoprolol, metropolol, propanolol, labetalol (alfa and beta) are approved for use by children and adolescents. The efficacy of metropolol was demonstrated in a clinical trial involving 140 children, showing to be effective in the 2 mg/kg dose [92]. Non-cardioselective agents should not be used in patients with asthma or heart failure, and their heart rate defines the dose to be used. These drugs can reduce the individual's performance during physical activity. These are not the most indicated drugs for diabetic patients using insulin as they favor hypoglycemia, unless under specific indications. Also, in individuals with insulin resistance, it has been shown that these agents increase the incidence of diabetes [93].

4. Diuretics such as hydrochlorothiazide, clortalidone, furosemide, espironolactone, trianterene and amiloride can be used. Thiazides and sub-products, as well as furosemide are more effective as anti-hypertensives than espironolactone and trianterene. Experience with adults has shown that the use of low doses of thiazides is effective as an antihypertensive. In association, diuretics have the ability to increase the efficiency of other antihypertensive agents which act by other mechanisms. Thiazides promote hypokalemia, an undesireable side effect, as it worsens glucose intolerance. This effect is dose-dependent and can be minimized if associated with a renin-angiotensin system blocker, an ARB or an ACEI, which favor potassium retention. Potassium-sparing diuretics are less effective and caution is necessary because when associated with ACEIs or ARBs, there is an increased chance of hyperkalemia. Furosemide can be indicated when edema is present, but can be useful in young patients with resistant hypertension, associated with other drugs, as well as in children with kidney failure.

5. Blockers of the sympathetic nervous system, peripheral alpha and beta-blockers and centrally acting alpha agonists such as clonidine, are considered in the treatment of resistant hypertension or when complications present, such as kidney failure. Clonidine causes side effects such as xerostomia, sedation and nasal obstruction. They are available in the American market in transdermic preparation, and their abrupt interruption could cause a hypertensive crisis due to an adrenergic discharge. Peripheral blockers like prazosin and doxasosin, are more widely used these days in association, and their advantage is the non alteration of the metabolic profile, as they even improve insulin sensitivity. The side effects are syncope, especially after the first dose and water retention. In the ALLHAT study, the use of prazozin was associated with negative endpoints and showed an increased incidence of heart failure.

6. Vasodilators like hydralazine and minoxidil are indicated when the blood pressure cannot be controlled, especially in children with kidney failure. These agents can cause tachycardia and water retention, and the concomitant use of diuretics may become necessary, especially in kidney failure, as well as the use of beta-blockers, for the reduction of heart rate. Hydralazine can cause lupus-like syndrome and the prolonged use of minoxidil can trigger hypertrichosis.

It can be concluded that anti-hypertensive treatment in children and adolescents should follow a customized plan according to the clinical condition and metabolic profile of the patient, having the reduction of cardiovascular morbimortality as the primary goal.

## Type 2 Diabetes in Children and Adolescents

The prevalence of type 2 diabetes mellitus (T2D) in children and adolescents has increased over the last 20 years in many countries and several ethnic groups [94]. In the USA, it has been indicated as an epidemic: 80% of Caucasian diabetic children over 10 years old had type 1 diabetes mellitus (T1D). On the other hand, 46%-86% of Afro-American, Hispanic, Asian-descendant or Amerindian-descendant children have been diagnosed with T2D [95]. In Europe and South America, the prevalence of T2D in children and adolescents is lower [96-99], but a German study showed that 2.5% of 700 obese school-age children presented impaired glucose tolerance (IGT) or diabetes [100], while an Argentinean study observed 1.6% of T2D and 7% of IGT in obese children [101]. In Japan, from 1976 to 1997, a ten-fold increase was observed in the presence of glucose abnormalities at the pediatric age range, a process which has been reverted through the adoption of public policies aiming at improving dietary habits [102].

In Brazil, there are few data on T2D prevalence in the young population. A study conducted in Fortaleza assessed 720 school-age children and found that capillary glucose levels were higher than normal in 8.3% of young people from 14 to 19 years old, most of whom were female [103]. At the childhood obesity clinic of The Group of Obesity in *Hospital das Clínicas *(University of Sao Paulo), abnormal fasting glucose levels and/or IGT were found, respectively, in 5% and 2.5% of the population studied (personal communication by Sandra M. F. Villares).

### Pathophysiology

The etiology of T2D in children and adolescents is multifactorial, similar to the one affecting adults, involving genetic and environmental factors; it results from the combination of insulin action resistance and β cell function failure. Insulin resistance is strongly associated with obesity, particularly central adiposity. This is believed to be the first abnormality in diabetes, preceding insulin secretion failure [104]. There is a 50% reduction in sensitivity and approximately 75% lower first phase insulin secretion in type 2 diabetic adolescents as compared to controls of non-diabetic obese individuals [105]. Insulin secretion failure seems to be more severe than the one observed in adults.

In adolescents at high risk of diabetes, the transition from normal tolerance to impaired glucose tolerance or pre-diabetes is associated with quick weight gain and a decrease in insulinogenic index, while the progression to T2D correlates to greater weight gain, reduced insulin sensitivity and a dramatic decrease in insulin secretion [106]. In type 2 diabetic adolescents, the decrease in β cell function, observed over 6 years of follow-up, was approximately 15% per year with no significant changes in insulin sensitivity [107]. Such loss of function is more than double and faster than the one observed in adults in the UKPDS study, which was 7% per year [108].

Additionally, T2D is commonly associated with other factors related to insulin resistance, such as hyperlipidemia, hypertension and non-alcoholic hepatic steatosis[109].

### Risk Factors for Type 2 Diabetes

The risk factors for T2D in young people include: family history, race and ethnicity, obesity and a sedentary lifestyle. The risk of developing T2D is 5 times higher for individuals with first-degree relatives with T2D as compared to controls of the same gender, age and weight with no family history of diabetes [110]. Determining factors for such risk are already present in fetal life.

The risk of metabolic syndrome during childhood was higher when neonates were on the weight curve extremities, i.e., large for gestational age (LGA) or small for gestational age (SGA). Similarly, children born from mothers with gestational diabetes mellitus have higher risk of developing T2D in adulthood [111,112].

In the USA, as previously described, T2D is two to six times more prevalent in African-American, Hispanic, Asian-descendant and Amerindian-descendant populations than in non-Hispanic Caucasians [113].

Several studies show that obesity associated with insulin resistance and T2D mellitus has largely increased in populations which have westernized their lifestyle, i.e., which have started consuming high-calorie diets and reduced physical activity [114].

Polycystic ovary syndrome (PCOS) and acanthosis nigricans are conditions associated with insulin resistance. A clinical study has shown that 30 to 32 % of young women between 14 and 19 years old with PCOS had impaired glucose tolerance [115,116]. Acanthosis nigricans, a disorder related with the activation of insulin receptors in the skin by insulin excess, is present in 90% of T2D children [115,117-119].

During puberty, there is a physiological peak of insulin resistance around Tanner stages 2 and 4, which returns to normality in early adulthood. Its peak occurred at Tanner 3 in both sexes, and girls were more insulin resistant than boys at all Tanner stages The diagnosis of T2D in young people occurs more frequently during the second decade of life, and the average age of diagnosis is 13.5, coinciding with the physiological peak of insulin resistance [29,31,120,121].

### Classification and Diagnosis of Diabetes in Childhood

The criteria for diagnosing diabetes in childhood are based on glucose levels and the presence of symptoms [122]. There are three possibilities for diagnosing diabetes, which, in the absence of unequivocal hyperglycemia symptoms, must be confirmed by dosages in subsequent days, as follows:

1. Fasting glycemia > 126 mg/dl,

2. Post-overload glucose levels with 1.75 g/kg of anhydrous glucose up to 75 g dissolved in water, ≥ 200 mg/dl

3. Classic symptoms of diabetes and casual glycemia ≥ 200 mg/dl, where 'casual' is defined as any time of day, not related to the last meal, and 'classic symptoms' include polyuria, polydipsia and unexplainable weight loss.

However, similarly as in adults, glycemic values often do not reach those established for diagnosing diabetes, but they are too elevated to be considered normal and must be classified as impaired fasting glucose levels (≥ 100 mg/dl, but < 126 mg/dl) or impaired glucose tolerance, 2 hours after glucose overload, glycemic levels above 140 mg/dl, but lower than 200 mg/dl.

On the other hand, images shown as type 1 diabetes (T1D) stereotypes - skinny children, with abrupt clinical condition - are characteristics that are no longer typical of the condition. Approximately one quarter of T1D patients may be obese at the moment of diagnosis. T1D may be confirmed by the 90% presence of anti-pancreatic antibodies at the moment of diagnosis, while the other 10% may have T2D, MODY or other types of diabetes (Tables 3 and 4).

**Table 3:** Classification of Diabetes Mellitus in Children and Adolescents

	T1D	T2D	ADM	MODY
Age at onset	Childhood	Puberty	Puberty	Puberty
Severity at onset	Acute/severe	Moderate to severe, usually insidious	Acute/severe	Mild/insidious
Insulin secretion	Very low	Varying	Moderately low	Varying
Insulin sensitivity*	Normal	Reduced	Normal	Normal
Insulin dependency**	Permanent	No	Varying	No
Genetic	Polygenic	Polygenic	Autosomal dominant	Autosomal dominant
Ethnic and racial distribution	All (less frequent in Asians)	Blacks, Hispanics, Asians and Amerindians	Blacks	Caucasians
Frequency (of all DM types in children and adolescents)	~80%	10 - 20%	5 - 10%	Rare
Association with Obesity	No	Strong	Varying	No
Acanthosis nigricans	No	Yes	No	No
Autoimmunity	Yes	No	No	No

DM: diabetes mellitus; T1D: type 1 diabetes mellitus; T2D: type 2 diabetes mellitus; ADM: atypical diabetes mellitus; MODY: maturity-onset diabetes of the young or monogenic diabetes.

* insulin sensitivity: considered a pathogenic factor. **insulin dependency is diagnosed in the absence of acute disease or other stress factors [119].

**Table 4:** Distinguishing features of type 1 and type 2 diabetes mellitus

	T1D	T2D
Start	Abrupt and symptomatic	Slow consolidation, with few symptoms
Family history	5% T1D	75-100% T2D
Insulin resistance	uncommon	common
Obesity	Obese, normal or thin	Typically present
Polidypsia, poliuria	symptomatic	Usually absent or mild
Ketoacidosis	30-40% at diagnosis	5-25 % at diagnosis
Hypertension and hyperlipemia	-	+
Sleep apnea	-	+
Acanthosis nigricans	-	+
Polycystic ovary	-	+
C-peptide levels	Low	Normal or elevated
Anti-pancreatic antibodies	+ (70-90%)	Absent
Acanthosis nigricans	-	+

T1D: type 1 diabetes; T2D: type 2 diabetes.

**Figure 1:**
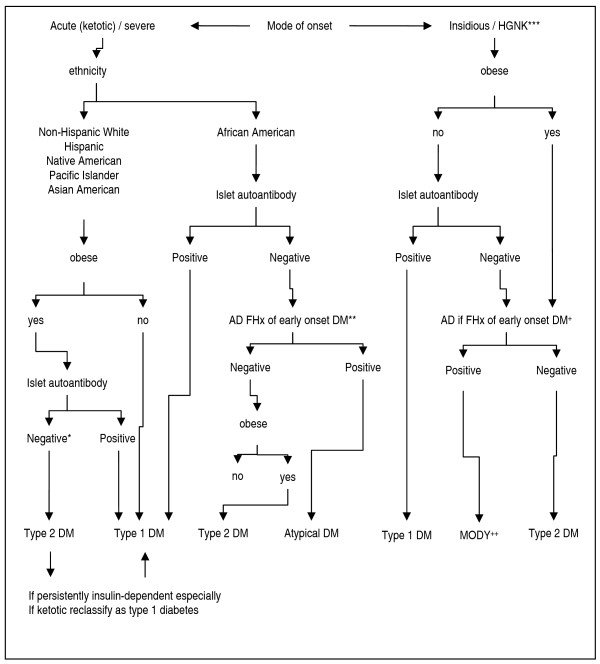
**Clinical classification of diabetes mellitus in children**. *No-specific autoimmunity; ** Autosomal dominant (AD) family history (FHx) of diabetes (DM) with an onset before 40 years of age;*** HGNK: hyperglycemia-nonketotic ^+^Autosomal dominant (AD) family history (FHx) of diabetes (DM) in more than three generations. ^++ ^Maturity-onset of diabetes of the young. Adapted from Rosenbloom, AL(127).

Research is recommended through antibodies dosage in overweight or obese children over 13 years old with clinical features of T1D mellitus, i.e., weight loss and ketosis or ketoacidosis (Figure 1).

Moreover, investigating the C-peptide levels is indicated for obese or overweight children over 13 years old, who have evolved with significant worsening of glycemic control during oral drug treatment. Plasma C-peptide levels over 1 ng/mL one year after diagnosis are highly suggestive of T2D [123,124].

It is recommended to assess all children and adolescents considered at risk of T2D (Table 5), from 10 years old or at early puberty stage, if first, and it must be repeated every two years. Fasting glycemia is the initial test recommended by the American Diabetes Association, due to its easy performance and convenience; even though data in literature show higher prevalence of impaired glucose tolerance and diabetes when glycemic curves are used for such assessments [123,125,126].

**Table 5:** Guidelines for investigating type 2 diabetes mellitus in children and adolescents with BMI > 85^th ^percentile for age and gender or weight for age, gender and height > 85^th ^percentile or weight > 120% of ideal weight for height (risk of obesity)

	Presence of at least two risk factors
Family History	T2D in 1^st ^and 2^nd ^degree relatives
Race/ethnicity*	American Indian, Black, Hispanic, Asian or Pacific Islander
Signals or conditions associated with insulin resistance	Acanthosis nigricans, arterial hypertension, dyslipidemia, polycystic ovary syndrome

Adapted: American Diabetes Association [123]. *In Brazil there are no data on race/ethnicity association as a risk factor for T2D, except for Asian adults.

### Clinical Condition of Children with T2D

Usually, typical T2D children or adolescents, similarly to adults, have few symptoms. There is also an intense family aggregation, as these children frequently have obese parents and first-degree relatives with T2D. Obesity may be predominantly visceral, with clinical features of metabolic syndrome and insulin resistance, such as: hypertension, hyperlipemia, sleep apnea, polycystic ovary syndrome and acanthosis nigricans. The initial condition of T2D in children or adolescents may be of hyperosmolar coma, currently known as the hyperglycemic dehydration syndrome [119]. This syndrome may affect up to 4% of the cases of T2D and presents mortality rates from 14% to 43% [127]. These children are as obese as adult type 2 diabetics, present change in level of consciousness (Glasgow scale on average from 9 to 15, but the impairment might be even more severe), average osmolarity of 400 mOsm and glycemic levels around 600 mg/dl. Usually, even after metabolic compensation, it may take from 1 to 7 days for the level of consciousness to return to normal [128-130]. In 5% to 50% of the cases, T2D may manifest initially as diabetic ketoacidosis [127,131].

### Treatment of Children with T2D

T2D treatment goals in children and adolescents are: reducing weight gain; maintaining linear growth; increasing physical activity; normalizing glucose levels and controlling comorbidities, including hypertension, dyslipidemia and hepatic steatosis.

After instituting non-pharmacological measures (diet and physical exercise), if results are not within the guideline targets for the specific age range, the introduction of metformin is recommended at half the dose usually prescribed to adults, increasing progressively up to 2/3 of the maximum dose recommended for the adult population [132,133]. Metformin decreases hepatic glucose production and increases glucose capture by peripheral tissues, as well as improving insulin resistance, body composition, insulinemia and, in type 2 diabetic adolescents with polycystic ovaries, improves SHBG and androgenic levels [42,44]. While prescribing it to adolescents with ovulation disorders, the need for contraception must be considered, as there is a possibility of inducing ovulation and unplanned pregnancy. Metformin reduces glycosylated hemoglobin by up to 1.2%, and abdominal pain is the most common side effect, affecting 25% of children [134,135].

If glycemic control is not achieved by lifestyle changes and metformin in monotherapy, the next therapeutic option would be sulfonylurea. It is known that these drugs increase both basal insulinemia as insulin secretion stimulated by food ingestion, leading to weight gain and higher risk of hypoglycemia [47]. The maximum dose recommended for children and adolescents corresponds to 2/3 of the maximum dose for adults and the reduction in glycosylated hemoglobin is approximately 0.8% [123]. Sulfonilureas (e.g., glibenclamida, glicazida, glimepiride) act by increasing both basal and meal-stimulated insulin secretion these obsviously carry the side effects of increasing weight gain and risk of hypoglycemia. There are no data regarding the appropriate management of type 2 diabetes in childhood.

Introducing insulin is indicated when the glucose level targets have not been reached through dietary changes, physical activity and oral agents in monotherapy or in association. However, evidence shows that introducing insulin early in the adult population makes long-term glycemic control easier, which may perhaps revert damage caused by hyperglycemia to β cells and insulin sensitivity [136-138]. Other medications employed in the T2D treatment of adults, such as glitazones, meglitinides, α-glucosidase inhibitor, amylin (pramlintide), incretinomimetic (exenatide) and DPP-4 inhibitors (gliptins) are not approved for use in pediatric populations.

Insulin is the longest-standing and most effective treatment for diabetes, because it is able to reduce glycosylated hemoglobin (HbA1c) to normal levels. However, the use of insulin is associated with weight gain (2-4 kg), probably related to glycemic level correction and subsequent reduction in glycosuria, and increased risk of hypoglycemia, due to high glycemic control intensification, targeting HbA1c levels lower than 7%. As advised by the latest ADA/EASD consensus [139], basal insulin may be added to oral drugs (metformin or metformin and sulfonylurea). Treatment is generally initiated by administering a medium-acting insulin (NPH) or long-acting insulin analog (levemir or glargine) dose of 0.1-0.2 U/kg of weight before bedtime, and insulin dose adjustments must be made according to the fasting capillary glycemic levels, measured on a daily basis. The insulin dose must be increased by 2-4 units every 3 days until reaching near-normal glycemic levels, with no unexplainable hypoglycemia.

After correcting fasting glycemia, glucose monitoring must be made at other times of the day, such as before dinner: if levels are proper, it is possible to infer that glycemic control goals have been reached. Another way of assessing the effectiveness of treatment is through the 8-point profile, i.e., measuring capillary glycemia before and after the 3 main meals, occasionally before bedtime and during the night, as well as fasting glycemia the following day, in order to identify moments when glycemic levels might be abnormal. It is suggested that measurements be made once a week or every 15 days, on different days of the week; however, this frequency may be individualized, depending on whether or not metabolic control is stable.

In the presence of hyperglycemia, when dieting errors and improper oral therapeutics are excluded, it is suggested to administer the second dose of NPH insulin before breakfast, which must be a 4-unit dose, and to maintain oral agents, progressively increasing the dose according to self-monitoring. However, a few patients may reach pre-prandial glucose targets and have hyperglycemia after meals, due to dieting errors or failure of oral agents indicated to control postprandial glucose levels. If dieting errors are corrected by keeping similar amounts of carbohydrates at each meal and postprandial hyperglycemia still occurs, it is suggested to introduce fast-acting or ultrafast-acting insulin before the "troublesome" meal, instead of an additional NPH insulin dose. In such case, fast-acting insulin before meals is recommended initially at a 4-unit dose, progressively increased based on postprandial glucose levels.

Diabetes mellitus is an evolutionary disease with progressive reduction in the number of β cells able to produce insulin, leading to insufficient secretion of the hormone and subsequent hyperglycemia, even in the presence of oral drugs and insulin therapy [140,141]. In this situation (beta-cell failure), intensive insulin therapy is indicated, where basal insulin (multiple doses of NPH or of long-acting analogs) is associated with fast-acting or ultrafast-acting insulin before the main meals. The therapy prescribed shall be basal-bolus insulin therapy, similar to the one recommended for treating T1D patients.

### Complications

In this age range, T2D evolves with more comorbidities than T1D [142,143]. Microvascular chronic complications, such as retinopathy, incipient nephropathy (microalbuminuria) and peripheral neuropathy, and macrovascular chronic complications are the most prevalent, and they decrease life expectancy and quality [143]. Table 6 shows the comparison made between young Australian populations under 18 years old with type 1 and T2D. In relation to the complication rate at diagnosis [142], results are similar to other populations [144].

**Table 6:** Chronic complication prevalence among type 1 and type 2 diabetes mellitus in children and adolescents

	T1D (%)	T2D (%)	p
Microalbuminuria	6	28	< 0.001
Arterial hypertension	16	36	< 0.001
Retinopathy	20	4	0.43
Peripheral neuropathy	27	21	0.48
Obesity	7	56	< 0.001

T1D: type 1 diabetes; T2D: type 2 diabetes. Adapted from Eppens, MC et al [142].

The prevalence of arterial hypertension and dyslipidemia is also much higher than in type 1 diabetic patients who have had the disease for the same period of time; a multicentric study conducted in the United States observed that arterial hypertension was present in 10-32% of young patients with T2D, and it is eight times more frequent than in T1D individuals of the same age [60]. Isolated or combined lipid changes were present in 24-44% of the cases, albeit only 1% of those children were undergoing pharmacological treatment with dyslipidemia [144].

Microalbuminuria is another important risk factor associated with T2D in children and adolescents, which gets worse over the period of time the patient has the disease [142,145-148]. Microalbuminuria is present in 14 - 25% at the moment of diagnosis; its incidence is high during the decade following the diagnosis and is linked to glycemic control. Additionally, microalbuminuria in T2D young individuals prematurely evolves to renal failure. During the follow up of Pima Indians with youth-onset type 2 diabetes mellitus, the incidence of diabetic end-stage renal disease was 5 times higher than compared to those who developed diabetes after the age of 20. Similar data were found in Japan when T2D diagnosis occurs before the age of 30 [149].

Apart from classic complications, the risk of non-alcoholic hepatic steatosis, hepatic cirrhosis and portal hypertension is five times higher in children with metabolic syndrome [150-152]. Neuropsychiatric diseases are also more prevalent in young type 2 diabetic individuals. Levitt-Katz and colleagues [153] have demonstrated that 20% of those patients had some neuropsychiatric disorder such as depression, attention deficit disorder, hyperactivity, neuropsychomotor development disorder, schizophrenia or bipolar disorder. Sixty three per cent of these patients were making use of antipsychotic drugs, increasing the possibility of weight gain by using such drugs, and the possibility of worsening metabolic control by using atypical antipsychotic drugs [154,155].

In conclusion, T2D in children and adolescents is associated with a high number of comorbidities and to the occurrence of complications, deeply affecting the quality of life of those individuals when they are still young. The proper treatment and especially the prevention must be attentively looked at by everyone involved in caring for children at risk of T2D mellitus.

## Competing interests

The authors declare that they have no competing interests.

## Authors' contributions

AH: wrote the section about Pharmacological Treatment; MM and MEM: wrote the section about Metabolic Syndrome Diagnosis in Children and Adolescents, revised the manuscript and made the requested formatting changes; MECM: wrote the section about Atherosclerosis in Young Adults; MF: wrote the section about Nutritional Orientation in Children and Adolescents with Metabolic Syndrome; RR: wrote the section about Physical Activity in Children and Adolescent; MCB and AB: wrote the section about Dyslipidemia; MTZ: wrote the section about Hypertension in Children and Adolescents; MQ and MN: wrote the section about T2D in Children and Adolescents.

All authors read and approved the final manuscript.
